# Biocompatibility Evaluation of Dental Luting Cements Using Cytokine Released from Human Oral Fibroblasts and Keratinocytes

**DOI:** 10.3390/ma8115372

**Published:** 2015-10-29

**Authors:** Jae-Sung Kwon, Yin-Zhu Piao, Sun-A Cho, Song-Yi Yang, Ji Hoon Kim, Susun An, Kwang-Mahn Kim

**Affiliations:** 1 BK21 PLUS Project, Department and Research Institute of Dental Biomaterials and Bioengineering, Yonsei University College of Dentistry, 50-1 Yonsei-ro, Seodaemun-gu, Seoul 120-752, Korea; jkwon@yuhs.ac (J.-S.K.); eunzoo0824@gmail.com (Y.-Z.P.); syyang88@yuhs.ac (S.-Y.Y.); 2 Safety Research Team/Skin Research Division, Amore-Pacific R&D Center, 1920 Yonggu-daero, Giheung-gu, Yongin-si, Gyeonggi-do 446-729, Korea; sunacho@amorepacific.com (S.-A.C.); jhkim84@amorepacific.com (J.H.K.); ssan@amorepacific.com (S.A.)

**Keywords:** biocompatibility, cytokine, cytotoxicity, dental luting cement, fibroblast, inflammation, keratinocyte

## Abstract

Dental luting cements are commonly used in dentistry for cementation of prosthetic restoration. Many previous studies focused on the measurement of the cell viability as the method of cytotoxicity evaluation during biocompatibility study for the material. In this study, the biocompatibility of various dental luting cements were evaluated using the new method of cytokine release measurement in order to better simulate inflammatory reactions in animal or clinical model using two different oral cells; immortalized human gingival fibroblast and immortalized human oral keratinocytes. Cells were exposed to extractions of various commercially available dental luting cements for different durations. Cytokines of IL-1α and IL-8 were measured from the supernatants of the cells and the results were then compared to the conventional MTT viability test. The result from the conventional cell viability study showed a relatively simple and straight forward indication that only one of the dental luting cements tested in this study was cytotoxic with increasing duration of exposure for both cells. Meanwhile, the result from the cytokine measurement study was much more complex at the time point they were measured, type of cells used for the study and the type of cytokines measured, all of which influenced the interpretation of the results. Hence, the better understanding of the cytokine release would be required for the application in biocompatibility evaluation.

## 1. Introduction

Dental luting cements are commonly used in dentistry for cementation of prosthetic restoration, for which the selection of the right cement is important to achieve long-term success of fixed restoration [1]. Various luting cements are currently available in the market, including glass ionomer cement (GIC), resin-modified glass ionomer cement (RMGIC), and resin-composite cement (RC), each with distinct characteristics [2].

GIC was introduced in early 1970s, has been used as a restorative material because of its fluoride-releasing properties, but has also been used as a luting cement because of its chemical property that allow its adhesion to the mineralized tissue [2,3]. RMGIC is a hybrid formulation between resin and glass ionomer components that retains the advantages of GIC, but also provides better setting characteristics and improved mechanical properties compared to the conventional GIC [4]. Finally, RC, which is polymerized by chemical initiation, photo initiation or a combination of both, has been used especially to meet esthetic demands [5].

Along with the above chemical and mechanical properties, biocompatibility of dental cement is also an important requirement that is essential for the acceptance of the materials [6]. It has been reported that some dental luting cements are cytotoxic to the dental pulp cells and cause hypersensitivity in animal studies [7]. There have also been numerous biocompatibility studies of dental luting cement using cell culture techniques with various cells, such as gingival fibroblasts [8], dental pulp cells [9,10] and mouse fibroblasts [11]. However, all of the previous studies focused on the viability of the cells as the method of cytotoxicity evaluation for dental cements, method which have often been criticized due to the poor correlation with either animal studies or clinical results, as it does not reflect inflammatory mechanisms of the body system [12].

Hence, the purpose of this study was to investigate the biocompatibility of various dental luting cements using both immortalized human oral fibroblasts and immortalized human oral keratinocytes by methods of cytokine release measurement. Two cytokines that have significant role in inflammatory mechanisms of the human body were considered in this study, IL-1α that plays a major role in regulating the release of other cytokines [13], and IL-8 that promotes wound healing through control of other cytokines and inducing growth factors expressions [14]. The results were then compared to the conventional viability test to suggest possible means of correlating the results. 

## 2. Experimental Section

### 2.1. Cells and Cell Culture

Two different human oral cells were used in this study; immortalized human gingival fibroblast (hTERT-hNOF) and immortalized human oral keratinocytes (IHOK). The hTERT-hNOF cells were derived from gingival fibroblasts that were primarily cultured from healthy human adults and transfected with puromycin-resistant retroviral vector plpc-hTERT (Clonetech Laboratories, Palo Alto, CA, USA). Previous studies have confirmed sub-culturing beyond the 90th passage without signs of replicative senescence and feasibility of biocompatibility evaluation has been shown [15,16].

IHOK were obtained from immortalization of human gingival epithelial cells by transfecting with the pLXN vector containing E6/E7 open reading frame of HPV type 16 as the method described previously [17].

Both cells were cultured in culture medium of Dulbecco’s Modified Eagle’s Medium mixed with Ham’s Nutrient Mixture F-12 in 3 to 1 ratio (DMEM/F12 3:1, Gibco, Grand Island, NY, USA). Additionally, 10% fetal bovine serum (Gibco, Grand Island, NY, USA) and 1% penicillin/streptomycin (Invitrogen, Grand Island, NY, USA) was added to the culture media and cells were cultured at 37 °C in a fully humidified atmosphere of 5% CO_2_. Despite the high calcium level of the media, and therefore not being optimal for the growth of IHOK, the selection of media was based on the consistency in extraction of the test materials as a different media would result in a different polarity, which would consequently cause different chemicals being extracted for the biocompatibility evaluations. In addition, it was confirmed that the DMEM/F12 3:1 media had no inhibition on growth of IHOK during the preliminary experiments (results not included) as well as in previously published studies [18].

### 2.2. Dental Luting Cements and Extraction

One of each commercially available GIC, RMGIC, and RC were evaluated in this study that was given with the code for the purpose of this manuscript (Table 1). All samples were checked for expiration dates and stored under manufacturers’ recommended conditions throughout the experiment.

Samples for extraction were made by mixing the material thoroughly according to the manufacturer’s instruction and placing each sample into a 16 mm diameter, 2 mm thick Teflon mold. The materials were then allowed to set, where, in case of RU, light curing was took place using a light-emission diode (LED) curing unit (Elipar S10, 3M ESPE, St. Paul, MN, USA) for 20 s on 5 different sides of the sample. 

Extracts from each type of cement were prepared according to the international standard and adaptated from a previous study [16,19,20]. Briefly, all samples were sterilized with ethylene oxide gas and extracted in serum free culture media where the volume was determined from the surface area of the samples (1.25 mL/cm^2^). Each type of cement was then extracted for 24 h at 37 °C. The serum free cell culture media without the sample was also incubated in the same condition as the test material extracts and used as the negative control of this experiment.

**Table 1:** Summary of dental luting cements used in this study.

Name	Code	Type	Batch No.	Manufacturer
Fuji I	FI	Glass ionomer cement	1207301	GC Corp., Tokyo, Japan
Fuji Plus	FP	Resin-modified glass ionomer cement	1209181	GC Corp., Tokyo, Japan
Rely X U200	RU	Resin cement	498329	3M ESPE, St. Paul, MN, USA

### 2.3. Cell Viability Test

A cell viability test was carried out according to international standards [21] using the MTT (3-(4,5-dimethylthiazol-2-yl)-2,5-diphenyl tetrazolium bromide) assay that generate formazan directly proportional to the number of viable cells by NADH-dependent cellular oxidoreductase enzyme of the mitochondria [22]. Briefly, 1 × 10^4^ of each cell was cultured in a standard 96-well plate (SPL, Gyeongi-do, Korea), in 100 µL of culture medium for 24 h. Following removal of the culture medium, and washing with Dulbecco’s phosphate buffer saline (Gibco, Grand Island, NY, USA), 100 µL of extractions from each type of cement (as described earlier) and negative control were placed on each cell. Cells were then incubated for 1.5 h, 3 h, 6 h, 12 h, 24 h, 36 h, 48 h, 60 h and 72 h at 37 °C and the supernatant from each cell was collected for cytokine release assay, which the method will be described below. Following removal of culture media, 50 µL of MTT solution (Sigma-Aldrich, St. Louis, MO, USA) was added to each well and incubated for further 2 h. MTT solution was then removed and 100 µL of isopropanol (Sigma-Aldrich, St. Louis, MO, USA) was added into each well that solubilize the formazan product. The optical density of each well was then measured using a microplate spectrophotometer (Epoch, BioTek Instrument, Winooski, VT, USA) at 570 nm following 1 h of shaking and the cell viability was calculated as the percentage of the optical density measured for the negative control.

### 2.4. Cytokine Release Test

Cell supernatant that was collected from above cell viability test was used for the cytokine release test. First, 50 µL of the supernatant from each of cells exposed to dental luting cement extracts or negative control were reacted with 50 µL of either mouse anti-human interleukin-1α (IL-1α) or interleukin-8 (IL-8) (R&D System Inc., Minneapolis, MN, USA) in standard 96-well plate for 2 h at room temperature. After the reaction, each well was washed three times with 0.05% Tween 20 in PBS (Sigma-Aldrich, St. Louis, MO, USA) and reacted with 50 µL of biotinylated goat anti-human IL-1α or IL-8 (R&D System Inc., Minneapolis, MN, USA). Each well was washed again with 0.05% Tween 20 in PBS (Sigma-Aldrich, St. Louis, MO, USA) and 50 µL of streptavidin conjugated to horseradish-peroxidase (R&D System Inc., Minneapolis, MN, USA) was added to each well. After final washing with 0.05% Tween 20 in PBS (Sigma-Aldrich, St. Louis, MO, USA), color was developed with tetramethylbenzidine (TMB, Sigma-Aldrich, St. Louis, MO, USA) and the reaction was stopped by 2 N H_2_SO_4_ (Sigma-Aldrich, St. Louis, MO, USA). The absorbance of each well was measured at 450 nm using a microplate spectrophotometer (Epoch, BioTek Instrument, Winooski, VT, USA) and levels of IL-1α and IL-8 were determined using calibrated curve drawn by dilutions of standard recombinant human IL-1α or IL-8 (R&D System Inc., Minneapolis, MN, USA).

### 2.5. Statistical Analysis

Each dental luting cement was tested with 8 wells of cultured cells for each time period of exposure and the entire experiment was repeated three times. The results of cell viability test and cytokine release assay were subjected to statistical analysis with the one-way ANOVA (PASW 18.0, IBM Co., Armonk, NY, USA), where Tukey method was adapted as a *post hoc* test, and the significance was declared at *p* < 0.05. All values were stated as mean ± standard deviations.

## 3. Results

### 3.1. Cell Viability Test

**Figure 1:**
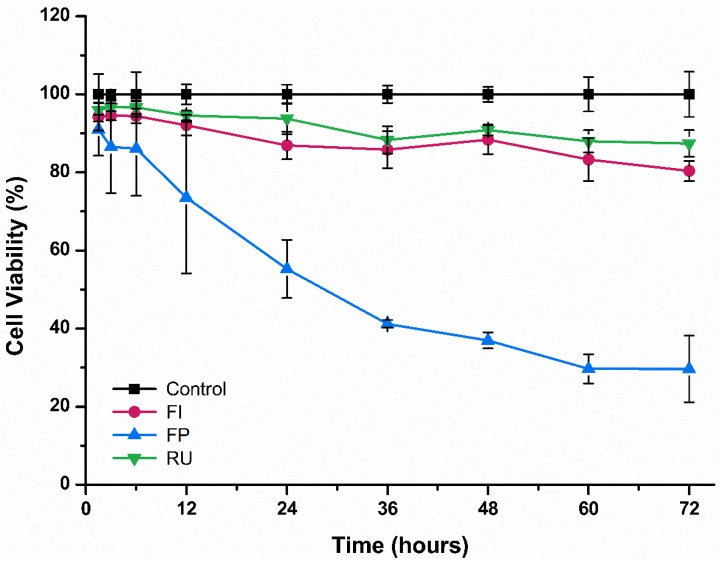
Cell viability of immortalized human oral keratinocytes (IHOK) following exposure to Fuji I (FI), Fuji Plus (FP) and Rely X U200 (RU) for varying duration of time.

**Figure 2:**
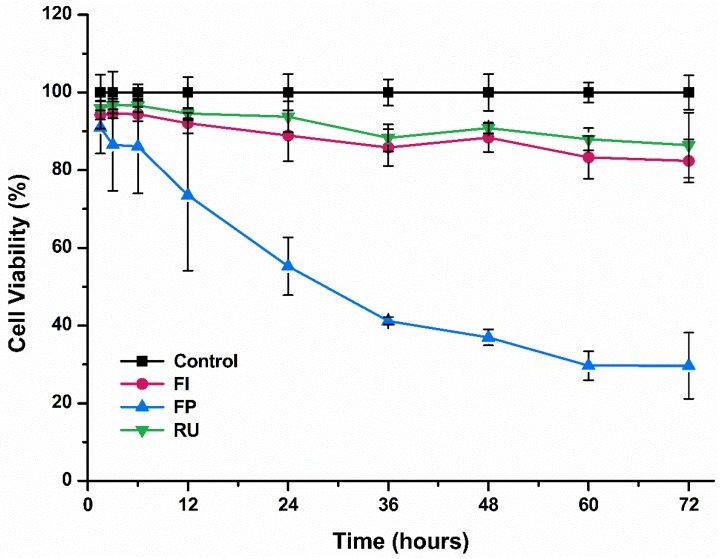
Cell viability of immortalized human oral fibroblasts (hTERT-hNOF) following exposure to Fuji I (FI), Fuji Plus (FP) and Rely X U200 (RU) for varying duration of time.

The results of cell viability test measured by MTT assay for three different types of dental luting cements are shown in Figure 1 and Figure 2. 

In terms of cell viability of IHOK, cells exposed to extraction of FP showed the lowest cell viability (*p* < 0.05) for each time period of exposure (1.5 h to 72 h) and there was a decrease in cell viability with the passage of time for FP (Figure 1). However, there was no significant difference between cells exposed to FI, RU and negative control (*p* > 0.05), and high level of cell viability was maintained for both FI and RU with the passage of time.

The results showed similar trend with cell viability of hTERT-hNOF following exposure to dental luting cements, though high level of cell viability was observed following short duration of exposure (1.5 h, 3 h, and 6 h) to extraction of FP (Figure 2).

### 3.2. Cytokine Release Test

**Figure 3:**
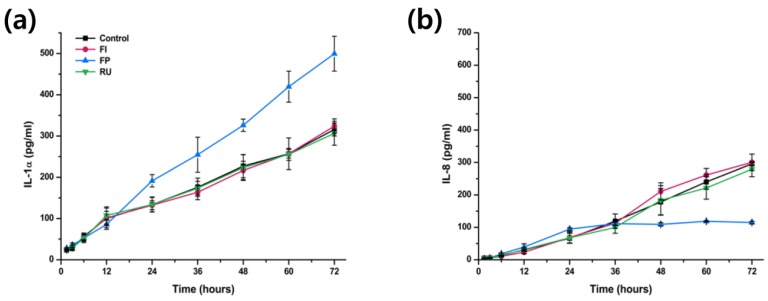
(**a**) Concentration of IL-1α and (**b**) IL-8 released from immortalized human oral keratinocytes (IHOK) following exposure to Fuji I (FI), Fuji Plus (FP) and Rely X U200 (RU) for varying duration of time.

**Figure 4:**
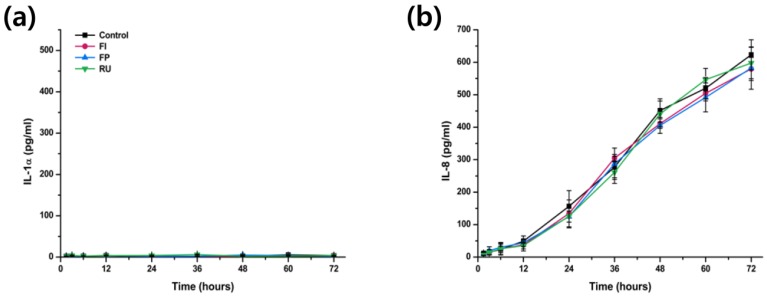
(**a**) Concentration of IL-1α and (**b**) IL-8 released from immortalized human oral fibroblasts (hTERT-hNOF) following exposure to Fuji I (FI), Fuji Plus (FP) and Rely X U200 (RU) for varying duration of time.

The results of cytokine release test for IL-1α and IL-8 from both IHOK and hTERT-hNOF following exposure to extraction of three different types of dental luting cements are shown in Figure 3 and Figure 4.

For IHOK, general increase of IL-1α release as the increasing duration of extraction exposure was noted for all of test and control group (Figure 3a). In addition, high level of IL-1α was detected following exposure to extraction of FP for 24 h or longer than any other dental luting cements (*p* < 0.05) and there was no significant difference in IL-1α level following exposure to FI, RU and negative control (*p* > 0.05) (Figure 3a).

The results for IL-8 released from IHOK were similar to IL-1α except for the IHOK exposed to extraction of FP. First, there was no significant difference among all test and control group until 12 h of exposure (*p* > 0.05) (Figure 3b). At the point of 24 h exposure, IHOK exposed to extraction of FP showed significantly higher level of IL-8 than FI, RU and negative control (*p* < 0.05). However, at 36 h of exposure, there was no significant difference between all of the test and control groups as before, and finally the IL-8 released from IHOK following exposure to extraction of FP showed significantly lower level of IL-8 compare to FI, RU and negative control from 48 h to 72 h of exposure (*p* < 0.05) (Figure 3b).

In terms of hTERT-hNOF, it was very difficult to detect IL-1α from any of the hTERT-hNOF exposed to dental luting cements and negative control (Figure 4a). There was, however, increasing level of IL-8 released from hTERT-hNOF at increasing exposure times to dental luting cements and negative control, and there was no significant difference between all of test and control groups (*p* > 0.05) (Figure 4b).

## 4. Discussion

Biocompatibility of dental luting cements is an important feature of the material, as with any dental materials, in order to avoid potential adverse biological effects when applied to patients in clinics [23]. In this study, three different types of dental luting cement, GIC, RMGIC and RC, were examined for *in vitro* cytotoxicity as part of biocompatibility evaluation using two different cell lines, hTERT-hNOF and IHOK.

First, a cell viability test using MTT assay was carried out, which showed that RMGIC tested in this study (FP) was significantly more cytotoxic than the other types of dental luting cements and than negative control for both cell types (Figure 1 and Figure 2). This result was in agreement with previous study that used human dental pulp stem cells that showed high level of cytotoxicity by FP due to excessive fluoride ion release and the presence of monomer in extracts [10]. Indeed, such cell viability test is useful in terms of evaluation of large number of materials and as indication of cellular metabolic activity [24,25]. However, it has often been suggested that careful consideration shall be given to such test, as it cannot reflect the tissue inflammatory responses that take place during both *in vivo* and clinical studies [12]. Hence, additional test that considered the inflammatory response was carried out in this study, the cytokine release test.

Initial interesting findings from cytokine release test was that there was no or very low level of IL-1α released from hTERT-hNOF cells. This result was somewhat in agreement with a previous study by Moharamzadeh *et al.* who showed that there was no IL-1β released from human gingival fibroblasts [26]. However, unlike their findings, which showed that HaCaT keratinocyte did not release any IL-1β either, the results in this study showed that IHOK managed to produce IL-1α from the extraction of dental luting cement, which may be due to the fact that different components of IL-1 was investigated (α instead of β) or due to the difference in sites where primary culture for immortalized cell line was obtained (skin tissue in HaCaT and oral tissue for IHOK).

In terms of the result of the other cytokine release test, significantly higher level of IL-1α was released from IHOK cells exposed to extraction of FP (Figure 3a), which was in agreement with cell viability test results. However, unlike the cell viability test, there was no significant difference in IL-8 released from any of the test and control materials for hTERT-hNOF cells (Figure 4b). Even more interestingly, significantly low level of IL-8 release was identified following exposure of FP extraction to IHOK for 48 h or longer (Figure 3b), which is a completely opposite trend to the cell viability test results. 

It is well known from previous *in vivo* animal studies and *ex vivo* organotypic tissue that oral mucosa cells are capable of producing both IL-1α and IL-8, even without any stimulation [27]. This was evident in this study as both cytokines were released (except IL-1α from hTERT-hNOF as mentioned above), even with exposure to negative control. In addition, oral mucosa keratinocytes have been known as the major source of IL-1α [27] and among many other roles of IL-1α, its major role is to regulate release of other cytokines such as IL-6 or IL-8 [13]. IL-8 then plays a role in promotion of wound healing through controlling other cytokines and inducing growth factors expressions [14]. Although the role and interaction between each cytokines are still unclear, the results seen in this study may have been due to the initial regulator of IL-1α that was released constantly by the presence of cytotoxic material, whereas the active component of IL-8 level may vary according to the time period depending on both IL-1α level and feedback from other cytokines. Still, it is expected that such variation of both cytokine level according to the duration of dental luting cement exposure would result in different response in animal studies compare to simple difference seen in cell viability results. In order to confirm the findings of this study and provide explanations for the results, molecular studies such as real-time quantitative reverse transcription polymerase chain reaction is currently underway for the IL-1α, IL-8, and other cytokines such as TNF-α, as the further investigations are outside the scope of this paper.

The interest toward the development of *in vitro* biocompatibility study that is an alternative to animal experiment is currently increasing, which the key issue is to simulate and predict biological reaction to materials when it is placed in the body through *in vitro* studies [28]. In this study, measurement of two cytokines, IL-1α and IL-8, was carried out on both keratinocytes and fibroblasts following exposure to different dental luting cements. The study has few limitations including use of DMEM/F12 3:1 media, which is not optimal for IHOK. Still, the results here showed the importance of considering three key factors during the measurement of cytokines as part of biocompatibility test: (1) consideration of cell type; (2) consideration of time point for measurement; and (3) consideration of cytokine type. These consideration and understandings would provide useful information in terms of applying method of cytokine measurement as *in vitro* biocompatibility study for any dental or biological materials. This would also provide foundation to the alternative test method for animal studies, by considering more clinically relevant *in vitro* biocompatibility study for the dental materials.

## 5. Conclusions

In this study, two cytokines, IL-1α and IL-8, were measured from two different cell lines, IHOK and hTERT-hNOF, following extraction of various commercially available dental luting cements to investigate possibilities of using them as method for biocompatibility evaluation. It was evident that type of cells used, type of cytokines measured and time point of measurement all influenced the interpretation of results. Understanding these three factors in link with conventional cell viability test studies as well as the animal or clinical studies would be required for better application of cytokine measurement for biocompatibility evaluation of biomaterial including dental luting cements.
